# Antiemetic effects of baclofen in a shrew model of postoperative nausea and vomiting: Whole‐transcriptome analysis in the nucleus of the solitary tract

**DOI:** 10.1111/cns.13823

**Published:** 2022-03-03

**Authors:** Daisuke Konno, Shigekazu Sugino, Tomoko F Shibata, Kazuharu Misawa, Yuka Imamura‐Kawasawa, Jun Suzuki, Kanta Kido, Masao Nagasaki, Masanori Yamauchi

**Affiliations:** ^1^ Department of Anesthesiology and Perioperative Medicine Tohoku University School of Medicine Sendai Japan; ^2^ Department of Integrative Genomics Tohoku University Tohoku Medical Megabank Organization Sendai Japan; ^3^ Department of Human Genetics Yokohama City University Graduate School of Medicine Yokohama Japan; ^4^ Department of Pharmacology Biochemistry and Molecular Biology Institute for Personalized Medicine Pennsylvania State University College of Medicine Hershey USA; ^5^ Department of Anesthesiology Kanagawa Dental University Graduate School of Dentistry Yokosuka Japan; ^6^ Center for the Promotion of Interdisciplinary Education and Research Kyoto University Kyoto Japan

**Keywords:** GABA_B_ receptor‐mediated signaling pathway, nucleus of the solitary tract, postoperative nausea and vomiting, whole‐transcriptome analysis

## Abstract

**Aims:**

The molecular genetic mechanisms underlying postoperative nausea and vomiting (PONV) in the brain have not been fully elucidated. This study aimed to determine the changes in whole transcriptome in the nucleus of the solitary tract (NTS) in an animal model of PONV, to screen a drug candidate and to elucidate the molecular genetic mechanisms of PONV development.

**Methods:**

Twenty‐one female musk shrews were assigned into three groups: the Surgery group (shrew PONV model, n = 9), the Sham group (n = 6), and the Naïve group (n = 6). In behavioral studies, the main outcome was the number of emetic episodes. In genetic experiments, changes in the transcriptome in the NTS were measured. In a separate study, 12 shrews were used to verify the candidate mechanism underlying PONV.

**Results:**

A median of six emetic episodes occurred in both the Sham and Surgery groups. Whole‐transcriptome analysis indicated the inhibition of the GABA_B_ receptor‐mediated signaling pathway in the PONV model. Baclofen (GABA_B_ receptor agonist) administration eliminated emetic behaviors in the shrew PONV model.

**Conclusions:**

Our findings suggest that the GABA_B_ receptor‐mediated signaling pathway is involved in emesis and that baclofen may be a novel therapeutic or prophylactic agent for PONV.

## Graphical abstract

Whole‐transcriptome analysis indicated the inhibition of the GABAB receptor‐mediated signaling pathway in the shrew PONV model. After the shrew emetic behaviors, the increase in ADCY1 gene expression, which translates adenyl cyclase (orange), the decrease in GABBR1 gene expression, which translates GABAB receptor R1 subunit (green), and the decrease in CACNB1 gene expression, which translates voltage‐gated calcium channel (blue), were observed. Pharmacological baclofen (GABAB receptor agonist) administration eliminated emetic behaviors in the shrew PONV model.

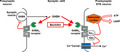

## 1 INTRODUCTION

Postoperative nausea and vomiting (PONV) is a serious and frequent complication in patients after they emerge from general anesthesia. Approximately 30% of all patients experience PONV, and in a subpopulation of high‐risk patients, the incidence increases to 80%.^1^ The latest guideline suggests that risk factors for PONV in adults are female sex, type of surgery (e.g., laparoscopic or gynecological surgery), volatile anesthesia, a history of motion sickness or PONV during previous surgeries, non‐smoking status, long surgery duration, younger age, and postoperative opioids.^2^ However, the central nervous system mechanisms that underlie the effects of these emetic stimuli on the development of PONV have not been fully elucidated.

Animal studies are rarely used in emetic research, in part because standard laboratory animals, such as rats and mice, are not able to vomit.^3^, ^4^ Although ferrets are most commonly used in emetic research, inhalation anesthetics do not induce nausea and vomiting in the ferrets.^5^ Musk shrews, *Suncus murinus*, are now being used in emetic research at several laboratories.^4^, ^6^, ^7^, ^8^, ^9^ Emetic behavior can be evoked in this small animal by various stimuli, including motion, nicotine, and inhalational anesthetics.^4^, ^6^, ^7^, ^8^, ^9^ Despite recent advances in shrew research, no studies have thoroughly examined how inhalation anesthetics and surgical insults influence the development of PONV in shrew models.

In the human brain, the nucleus of the solitary tract (NTS) in the brainstem converges the primary afferents by emetic stimulation from the vestibular system, abdominal vagal afferents, and the area postrema; sends outputs to the somatosensory/viscerosensory cortex via the parabrachial nucleus and the thalamus for the induction of nausea sensation; and sends outputs to the gastrointestinal system and the respiratory system for induction of retching or vomiting. Previous anatomical and physiological studies have indicated that the NTS plays a central role in processing emetic information.^3^ The aims of this study were to determine changes in genome‐wide gene expression (i.e., whole‐transcriptome analysis) in the NTS in a musk shrew PONV model and elucidate the molecular genetic mechanisms of PONV development.

## 2 MATERIALS AND METHODS

### 2.1 Animals

This study was approved by the Tohoku University Institutional Animal Care and Use Committee (#2017‐236). No specific inclusion or exclusion criteria were set. In total, 33 female musk shrews were used (Jic:SUN‐Her/Kwl strain, aged 7–10 weeks, weighing 30–50 g). The animals were housed individually in 35 cm × 30 cm × 17 cm plastic cages on soft bedding, maintained at 22 ± 2°C with a 12‐h light/dark cycle, and provided with trout pellets and tap water *ad libitum*. Efforts were made to reduce both animal numbers and suffering during the experiments. This research was reported in accordance with the ARRIVE guideline.^10^

### 2.2 Low abdominal surgical procedure

Laparotomy was performed as described previously, with slight modification.^11^, ^12^ Briefly, the lower abdomen of the shrew was shaved and disinfected with povidone‐iodine, and a 1‐cm vertical incision was made under 5% isoflurane anesthesia in 1 L/min of oxygen, administered via a facemask. Intestinal paralysis was induced by manipulating the small intestine vigorously with a cotton swab for 30 s. The abdominal muscle and skin were then closed, applying three sutures to each layer using 3–0 braided nylon sutures (Surgilon; Covidien Ltd., Minneapolis, MN). The entire surgical procedure was completed within 10 min.

### 2.3 Experimental groups

To determine the effects of isoflurane and low abdominal surgery on shrews, the animals were randomly assigned to three groups: the Surgery group (shrew PONV model, n = 9), treated by incision and suturing of the lower abdomen under 5% of isoflurane inhalation, as described above; the Sham group (n = 6), treated only by shaving the lower abdomen under 5% isoflurane inhalation for 10 min; and the Naïve group (n = 6), which received no treatment.

In a separate study, 12 shrews were randomly assigned to two groups and treated as follows: the Baclofen group (n = 6), lower abdominal surgery followed by single‐dose 5 mg/kg intraperitoneal injection of baclofen (a GABA_B_ receptor agonist); and the Vehicle group (n = 6), surgery followed by intraperitoneal injection of normal saline alone. The doses of drugs were determined in preliminary experiments based on previous reports.^13^, ^14^ Baclofen or normal saline was administered at 3 min before the start of the emetic behavioral test.

### 2.4 Emetic behavioral test protocol

The shrews were housed in the institutional rearing area for more than 7 days, and each shrew was transported from the home cage to an observation chamber (cylinder‐shaped, 20 cm in diameter ×30 cm in height, made from clear acrylic), where it was allowed free movement for habituation for 30 min. The shrew was then placed in a transparent induction chamber (box‐shaped, 15 cm × 10 cm × 10 cm). Shrews in the Surgery or Sham group were anesthetized by isoflurane in 3 L/min of oxygen, with the concentration of isoflurane increased stepwise from 1% to 4% in increments of 1% during 5 min. After the withdrawal of reflexes to pinching of the tail and the hind paws, the shrew was placed on a surgical table under 5% isoflurane anesthesia with 1 L/min of oxygen administered via a facemask. Shrews in the Surgery group then underwent the surgical procedure described above, whereas those in the Sham group received only hair shaving and disinfection of the lower abdomen. In the Naïve group, the shrews were exposed to 3 L/min of oxygen in the induction chamber without isoflurane or surgery.

After the treatment, the shrew was transferred back to the observation chamber. Emetic episodes were characterized by rhythmic abdominal contractions that were either associated with the oral expulsion of solid or liquid material (i.e., vomiting) or not associated with the passage of material (i.e., retching). Emetic episodes were counted as separate episodes when the interval between vomiting and/or retching exceeded 2 s. The emetic episodes were counted in real time during anesthetic emergence in the observation chamber for 30 min after the treatment. Latencies to the first emetic episode were measured during anesthetic emergence after the transfer to the observation chamber. Times from the first episode to the last episode in the observation chamber were also measured. Latencies to the start of the walk were measured during anesthetic emergence after the transfer to the observation chamber. The emetic episodes in the observation chamber were later verified by viewing recordings from two video cameras positioned in the upper front sides of the observation chamber, with two mirrors positioned at the rear of the chamber, so that the shrew’s behavior could be seen from all directions.

### 2.5 NTS isolation followed by total RNA extraction

At 1 h after the start of exposure to oxygen with or without isoflurane, each shrew was euthanized via CO_2_ exposure. The shrews were rapidly decapitated with a laboratory guillotine, and the brain stem was immediately removed within 10 min. According to the stereotaxic atlas of the rat brain and shrew,^15^ the NTS was dissected from a frozen section of the brain stem, and total RNA was extracted from NTS using NucleoSpin RNA/Protein Kit (Macherey‐Nagel, Düren, Germany).

### 2.6 Whole‐transcriptome sequencing (RNA‐seq)

The density of extracted RNAs was measured using a Qubit3.0 Fluorometer (Thermo Fisher Scientific, Waltham, MA). Whole‐transcriptome sequencing was then performed from three RNA samples in the Surgery group, three RNA samples in the Sham group, and three RNA samples in the Naïve group by using a next‐generation DNA sequencer (Illumina HiSeq 2500). The cDNA libraries were prepared using the NEXTflex™ Illumina Rapid Directional RNA‐Seq Library Prep Kit (BioO Scientific, Austin, TX) as per the manufacturer’s instructions. Briefly, polyA RNA was purified from 200 ng of total RNA using oligo (dT) beads. The extracted mRNA fraction was subjected to fragmentation, reverse transcription, end repair, 3’‐end adenylation, and adaptor ligation, followed by PCR amplification and SPRI bead purification (Beckman Coulter, Brea, CA). The unique index sequences were incorporated in the adaptors for multiplexed high‐throughput sequencing. The final product was assessed for its size distribution and concentration using BioAnalyzer High Sensitivity DNA Kit (Agilent Technologies). Pooled libraries were diluted to 2 nM in EB buffer (Qiagen, Hidden, Germany) and then denatured using the Illumina protocol. The denatured libraries were diluted to 10 pM by pre‐chilled hybridization buffer and loaded onto a TruSeq v2 Rapid flow cell on an Illumina HiSeq 2500 and run for 50 cycles using a paired‐read recipe according to the manufacturer’s instructions.

### 2.7 Computational bioinformatic analysis

The RNA‐seq reads were checked for quality by using FastQC ver. 0.11.4. These reads were mapped to the shrew reference genome (Ous:KAT‐227c strain, CDS +UTR sequence, Suncus murinus Genome Project in Japan, unpublished draft sequence) by using Bowtie2 ver. 2.3.4.1.^16^ The mapped reads were assembled and annotated by using TIGAR2 ver. 2.1 supplied by Suncus murinus Genome Project protein‐coding gene annotation file.^17^ Differential expressed transcripts (DEX) were compared between the two selected groups by using edgeR ver. 3.5.0.^18^ Significance was defined as results with a *q* value of less than 0.05 calculated by the Benjamini–Hochberg method to control the false discovery rate (FDR). MA plots were generated by using the plotSmear function of edgeR software.

With respect to the DEX between the Surgery and Naïve groups (see the Results section), gene symbol names of top‐100 differentially expressed transcripts with fold changes and *p* values were analyzed with ingenuity pathway analysis (IPA, version 60467501, release date: Nov 11, 2020, Qiagen). Duplicated gene symbol names were excluded to avoid the co‐existence of the upregulated and downregulated transcripts with the same symbol name. Significance was determined using the right‐tailed Fisher’s exact test (*p* < 0.01) according to the manufacturer’s instructions.^19^ To decipher possible functions of the genes, the gene symbol names with statistical significance in IPA were analyzed by web‐based gene ontology enrichment analysis (g:GOSt, https://biit.cs.ut.ee/gprofiler).^20^ Functional information for humans was used, and significance was determined using the g:SCS algorithm (*q* < 0.01).

### 2.8 Statistical analyses

Statistical analyses were performed with EZR (Saitama Medical Centre, Jichi Medical University, Saitama, Japan), which is a graphical user interface for R (The R Foundation for Statistical Computing, Vienna, Austria) modified to add statistical functions. Normality in continuous variables, reported as medians (interquartile ranges) unless otherwise noted, was tested using one‐sample Kolmogorov–Smirnov tests. Differences in continuous variables were tested for significance by the Mann–Whitney test or the Kruskal–Wallis test with the Steel–Dwass test for multiple comparisons. All *p* values were two‐sided, and *p* < 0.05 was considered to indicate statistical significance.

## 3 RESULTS

### 3.1 In the behavioral study, isoflurane alone, or surgical insult under isoflurane anesthesia, induced emesis in shrews

**FIGURE 1:**
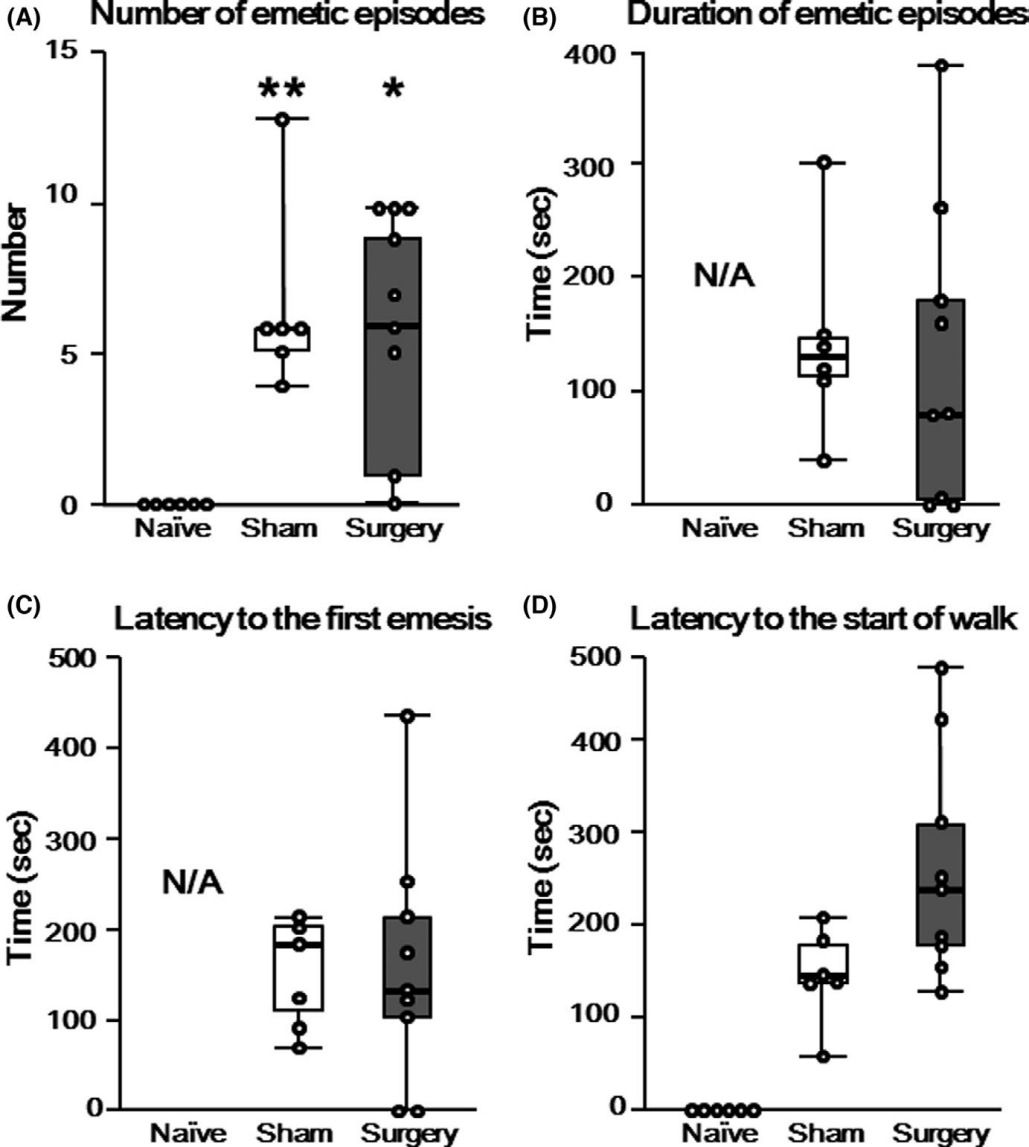
(A–D) Emetic behaviors of the shrews. (A) Number of emetic behaviors. (B) Duration of emetic behaviors. (C) Latency to the first emetic behavior. (D) Latency to the start of walking. Emetic episodes occurred in the Sham and Surgery groups. The bee‐swarm (dot) plot represents the measured values. The box represents the median and 25th–75th percentiles. Error bars represent the maximum or minimum. *p < 0.05 versus Naïve group. **p < 0.01 versus Naïve group

We developed a shrew PONV model in the current study (see Supplemental Movie [6 min 14 s] Supplementary Files). Figure 1A–D shows measurements of emetic episodes in the Naïve, Sham, and Surgery groups. All measurement values showed a non‐Gaussian distribution. The emetic episodes never occurred among the shrews in the Naïve group (Figure 1A). There were 6 (1) emetic episodes in the Sham group (Figure 1A) and 6 (8) emetic episodes in the Surgery group (Figure 1A). The numbers of emetic episodes of shrews in the Surgery and Sham groups were significantly larger than that in the Naïve group, but there were no differences between the Surgery group and the Sham group [*p* = 0.007, post hoc; *p* = 0.02 (Surgery vs. Naïve), *p* = 0.005 (Sham vs. Naïve), *p* = 0.97 (Surgery vs. Sham)]. The durations of all emetic episodes in the Sham and Surgery groups were 130 (35) s and 80 (175) s, respectively (Figure 1B), and there were no differences between the two groups (*p* = 0.68). The latencies to the first emetic episodes in the Sham and Surgery groups were 180 (87.5) and 130 (105) seconds, respectively (Figure 1C), and there was no difference between the two groups (*p* = 0.95). The latencies to the start of the walk in the Sham and Surgery groups were 145 (40) and 240 (130) seconds, respectively (Figure 1D), and there was no difference between the two groups (*p* = 0.06).

### 3.2 Whole‐transcriptome sequencing indicated that surgical insult under isoflurane anesthesia inhibited the GABA_B_ receptor‐mediated signaling pathway in shrews

**FIGURE 2:**
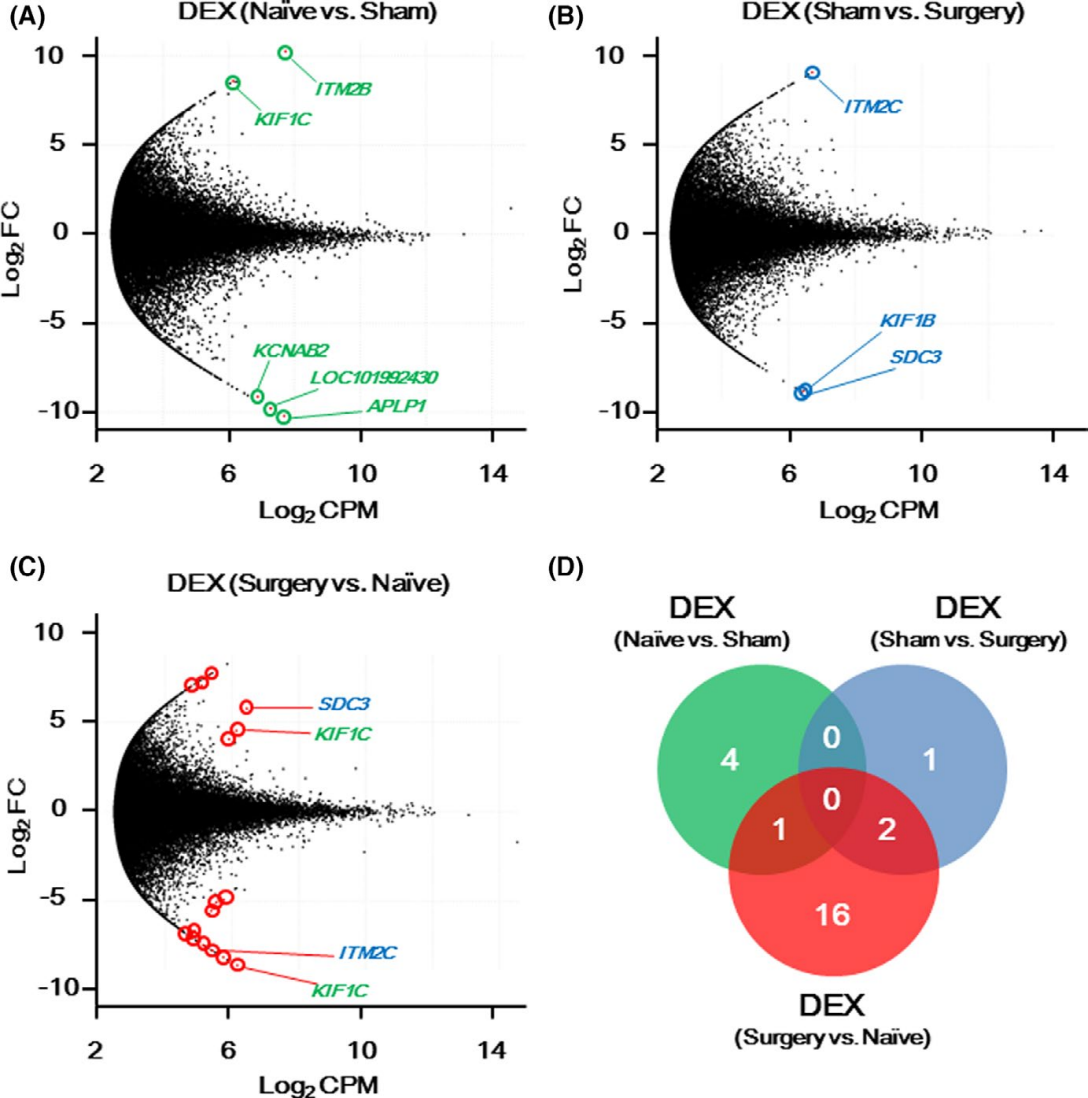
(A–C) MA plots of transcripts in the nucleus of the solitary tract. Red scatter with colored circle represents differentially expressed transcripts (DEX) with a false discovery rate <0.05. (A) Five highly differentially expressed genes were identified between the Naïve and Sham groups (green gene symbol names). (B) Three highly differentially expressed genes were identified between the Sham and Surgery groups (blue gene symbol names). (C) Nineteen highly differentially expressed genes were identified between the Naïve and Surgery groups. The *KIF1C* gene and its two transcripts were common among the DEX between the Naïve and Sham groups (green gene symbol names). The *SDC3* and *ITM2C* genes were common among the DEX between the Sham and Surgery groups (blue gene symbol names). (D) Venn diagram representing the number of significantly differentially expressed genes across three DEXs. Log_2_ FC: logarithm of fold change between the two groups, Log_2_ CPM: average logarithm of the counts per million

To examine the molecular genetic mechanism of the development of PONV, RNA‐seq was performed in RNA samples from three in the Surgery group, three in the Sham group, and three in the Naïve group. The concentration of the extracted RNAs was 38.6 (24.6) ng/μL. In total, 851,445,648 paired‐end reads (100 bp) were sequenced, and 59.7% of the reads were mapped to the shrew reference genome. The expression levels of 52,381 transcripts were identified in the three groups. Figure 2A–C shows three comparisons across the Naïve, the Sham, and the Surgery groups, and shows the DEXs in each comparison. The *ITM2B*, *KIF1C*, *KCNAB2*, *LOC101992430*, and *APLP1* genes were identified as DEX between the Naïve and Sham groups (Figure 2A). The *ITM2C*, *KIF1B*, and *SDC3* genes were identified as DEX between the Sham and the Surgery groups (Figure 2B). We identified 19 differentially expressed transcripts, including *SDC3*, *KIF1C*, and *ITM2C* genes, among the Naïve and Surgery groups (Figure 2C). As shown in Figure 2D, the highest DEX existed between the Surgery and the Naïve groups.

**FIGURE 3:**
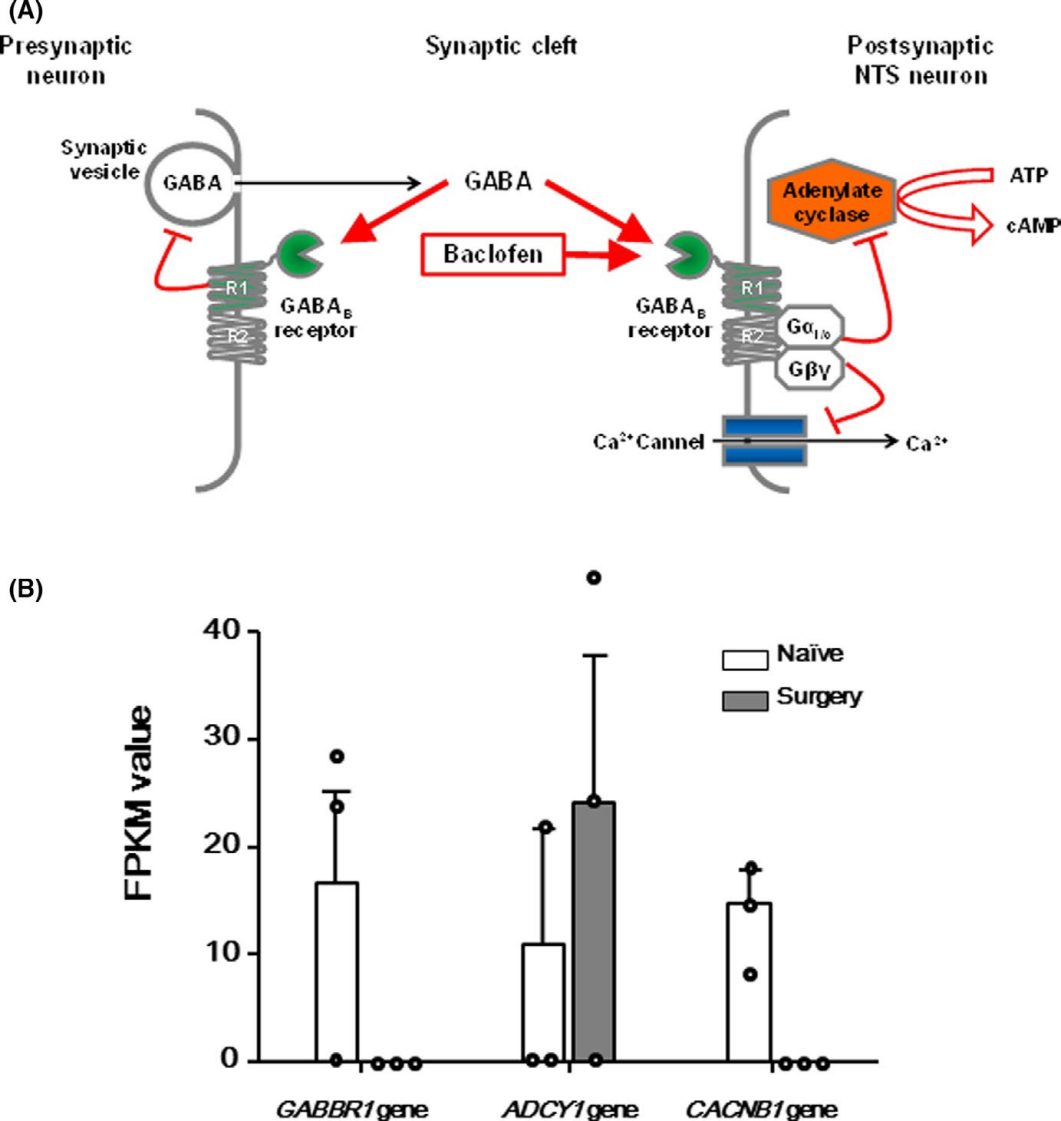
(A) Schematic drawing of inhibition of the GABA_B_ receptor‐mediated signaling pathway in the shrew PONV model. In shrews showing emetic behaviors, expression of the *ADCY1* gene, which encodes adenyl cyclase (orange), was increased, whereas expression of the *GABBR1* and *CACNB1* genes, which encode GABAB receptor R1 subunit (green) and voltage‐gated calcium channel (blue), was decreased. Administration of pharmacological baclofen (a GABA_B_ receptor agonist) eliminated emetic behaviors in the shrew PONV model. (B) Expression levels of the *GABBR1*, *ADCY1*, and *CACNB1* genes in whole‐transcriptome sequencing. Bar graphs and error bars indicate means and standard errors of the means. The gene expression of *GABBR1* and *CACNB1* was not observed in the Surgery group. FPKM value: fragments per kilobase of exon per million mapped fragments for quantifying the assembled transcript expression

We focused on the combined effects of surgical insult and inhalation anesthetic on PONV, which may be important in the clinical setting because general anesthesia is not applied to patients alone without another procedure, such as surgery. To identify canonical biological pathways and upstream regulators associated with the development of PONV after surgical insult under isoflurane anesthesia, 92 gene symbol names of the top‐100 highest differentially expressed transcripts (*p* < 0.001, supplemental table) between the Surgery and Naïve groups were then analyzed by using IPA. The IPA identified six pathways and 10 genes (Table 1, all *p* < 0.01). The gene ontology enrichment analysis revealed that these 10 genes were significantly enriched to 21 GO terms: three GO molecular function; 12 GO biological processes, including the G protein‐coupled receptor signaling pathway, which annotated *ADCY1*, *GABBR1*, and *HTR7* genes; and six cellular components regarding cell membrane location (Table 2, all *q* < 0.01). We ultimately chose the GABA_B_ receptor‐mediated signaling pathway and focused on GABA_B_ receptor, adenylyl cyclase, and voltage‐dependent calcium channel as candidate molecules associated with PONV. Figure 3 summarized *GABBR1*, *ADCY1*, and *CACNB1* gene expression levels in the pathway from the results of RNA‐seq in the shrew NTS (Figure 3A and B).

**TABLE 1:** PONV‐associated biological pathways and potential upstream regulators

Biological pathway	Gene	*p* value
cAMP‐mediated signaling	*ADCY1*, *CAMK1G*, *GABBR1*, *HTR7*, *TDP2*	0.002
Adenine and adenosine salvage VI	*ADK*	0.004
G protein‐coupled receptor signaling	*ADCY1*, *AKT2*, *GABBR1*, *HTR7*, *TDP2*	0.004
GABA receptor signaling	*ADCY1*, *CACNB1*, *GABBR1*	0.006
Pancreatic adenocarcinoma signaling	*AKT2*, *HMOX1*, *RALGDS*	0.008
Role of NFAT in cardiac hypertrophy	*ADCY1*, *AKT2*, *CACNB1*, *CAMK1G*	0.009

Potential upstream regulators	Regulated gene	*p* value
APP	*ALDOC*, *ANXA6*, *BACE1*, *HMOX1*, *PFKFB3*, *RASSF2*, *SAP30*, *TTR*	0.033
XB1	*ALG12*, *GMPPA*, *HMOX1*, *TTR*	0.013
NUPR1	*ALDOC*, *GABBR1*, *NCKIPSD*, *PFKFB3*, *SIPA1L2*, *TDRKH*	0.014

**TABLE 2:** Gene ontology enrichment analysis in 10 genes

GO molecular function	ID	Number of annotated genes	*q* value
Binding	GO:0005488	10/10	0.0008
Molecular function	GO:0003674	10/10	0.002
Protein binding	GO:0005515	9/10	0.005
GO biological process			
Signaling	GO:0023052	8/10	0.0007
Cell communication	GO:0007154	8/10	0.0007
Cellular response to stimulus	GO:0051716	8/10	0.002
Cellular response to organonitrogen compound	GO:0071417	4/10	0.002
Cellular process	GO:0009987	10/10	0.002
Response to endogenous stimulus	GO:0009719	5/10	0.003
Cellular response to nitrogen compound	GO:1901699	4/10	0.003
Response to oxygen‐containing compound	GO:1901700	5/10	0.003
Biological process	GO:0008150	10/10	0.004
G protein‐coupled receptor signaling pathway	GO:0007187	3/10	0.007
Response to stimulus	GO:0050896	8/10	0.009
Signal transduction	GO:0007165	7/10	0.009
GO cellular component			
Plasma membrane	GO:0005886	7/10	0.001
Cell periphery	GO:0071944	7/10	0.001
Cellular anatomical entity	GO:0110165	10/10	0.001
Cellular component	GO:0005575	10/10	0.002
Plasma membrane region	GO:0098590	4/10	0.006
Synapse	GO:0045202	4/10	0.007

Note

GO, gene ontology. Number of annotated genes: number of annotated genes of the 10 target genes in each GO term.

### 3.3 GABA_B_ receptor agonism produced the elimination of emetic behaviors in shrews

**FIGURE 4:**
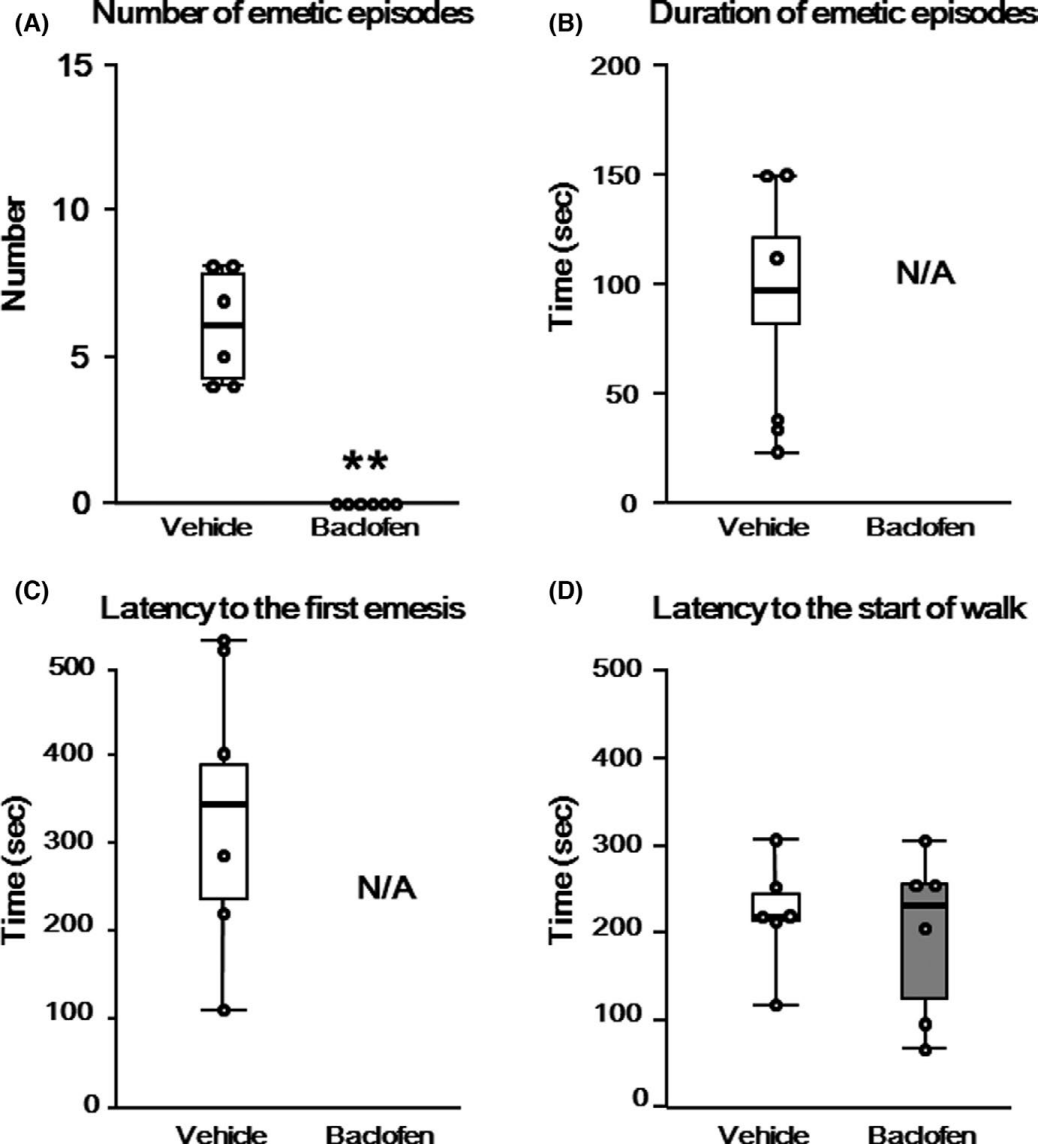
(A–D) Emetic behaviors of the shrews. (A) Number of emetic behaviors. (B) Duration of emetic behaviors. (C) Latency to the first emetic behavior. (D) Latency to the start of walking. Emetic episodes were eliminated in the Baclofen group. The bee‐swarm (dot) plot represents the measured values. The box represents the median and 25th to 75th percentiles. Error bars represent the maximum or minimum. *p < 0.05 versus the Naïve group. **p < 0.01 versus the Naïve group. N/A: not applicable

We hypothesized that administration of baclofen, a GABA_B_ receptor agonist, decreased the numbers of emetic episodes via activation of the GABA_B_ receptor‐mediated signaling pathway in the female shrew PONV model. In another series of behavioral experiments using 12 shrews, treatment with baclofen completely abolished the numbers of the emetic episodes in the Baclofen group in the shrew PONV model [Figure 4A, 0 (0) vs. 6(4), *p* = 0.003]. In the Vehicle group, the duration of all emetic episodes was 95 (83) s (Figure 4B). The latency to the first emetic episode was 343 (254) s (Figure 4C). The latencies to the start of the walk in the Vehicle and Baclofen groups were 225 (34) and 250 (160) seconds, respectively, and there was no difference between the two groups (Figure 4D, *p* = 1.00). All measurement values showed a non‐Gaussian distribution.

## 4 DISCUSSION

In the current study, we first developed a shrew model of PONV. The model used female musk shrews and applied surgical insult to the lower abdomen; thus, this model could be used to investigate PONV after gynecological surgery, such as hysterectomy or oophorectomy, and may be quite useful for preclinical emetic research.^21^ Next, we identified decreased *GABBR1* gene expression level, increased *ADCY1* gene expression, and decreased *CACNB1* gene expression in the NTS of the shrew PONV model using whole‐transcriptome sequencing followed by pathway and gene annotation analyses. We hypothesized that the GABA_B_ receptor‐mediated signaling pathway may be involved in the development of emesis in the shrew NTS. We finally revealed that systematic administration of baclofen, an agonist of the GABA_B_ receptor, significantly decreased the number of emetic episodes in the shrew PONV model.

In the present behavioral study, we used 5% isoflurane for abdominal surgery to acquire surgical anesthetic depth. With respect to the concentration of isoflurane, the minimum alveolar concentration (MAC) of isoflurane was determined in shrews using the tail‐clamp technique before the current study. In this preliminary experiment, the MAC value for 50% shrew immobility was 2.83% (2.5–3.0%, n = 3, data not shown). This MAC value in shrews was higher than that in other laboratory animals, such as rats and mice.^22^, ^23^, ^24^ In a single species, the variation in the MAC values is generally small. Even between species, the variation is not usually large. Interspecies differences in the profiles of spinal alpha‐motor neurons showed smaller anesthetic potency than that in rodents. We used 1.77 MAC (2.83/5) of isoflurane for abdominal surgery. This anesthetic depth prevents the sympathetic reflex in 50% of subjects in response to noxious stimuli.^25^

The frequencies of emetic episodes were similar in the Sham and Surgery groups (see the equal medians as shown in Figure 1). Nevertheless, in the Surgery group, the frequencies varied and tended to decreased, as demonstrated by the height of the box in the bar for the Surgery group in Figure 1. Surgical insult may be a preventable factor for emesis of shrews. In humans, type of surgery (e.g., laparoscopic surgery) and duration of surgery are risk factors for PONV in adults.^2^ The results in the current behavioral study conflicted with clinical observations. However, the association of surgical insult with PONV remains unknown.

In RNA‐seq analysis, changes in the transcriptome after isoflurane anesthesia (Figure 2A) and after isoflurane anesthesia followed by surgery (Figure 2B) overlapped for a few genes, that is, *SDC3*, *KIF1C*, and *ITM2C* (Figure 2C). The *SDC3* gene encodes syndecan protein, which is involved in the cytoskeleton structure. The *KIF1C* gene encodes kinesin proteins, which function as a cell microtubule‐dependent molecular motor. The *ITM2C* gene encodes an integral membrane protein that functions as a regulator for amyloid‐β protein. Although syndecan protein may be involved in obesity or appetite, the associations of all three genes with emesis are still unknown.^26^, ^27^

For DEX between the Naïve and Surgery groups, the other 15 genes, except for *SDC3*, *KIF1C*, and *ITM2C* genes, were highly expressed with statistical significance (Figure 2C, red dots with red circles). This observation suggested that surgical insult and inhalation anesthetics were not independent factors. As described above, gynecological surgery under general anesthesia, which is modeled by the Surgery group in this study, is a significant risk factor for PONV in clinical situations.^2^ Taking into consideration clinical settings, the downstream analysis focused on the changes in the whole‐transcriptome in the NTS between the Naïve and Surgery groups.

The following computational analysis using the IPA indicated the associations of the six biological pathways, 10 genes, and three upstream regulators (Table 1). Also, the gene ontology enrichment analysis showed a total of 21 GO terms (Table 2). We interpreted ubiquitous terms, such as “Protein binding (GO:0005515)” or “Cellular response to stimulus (GO:0051716),” as nonspecific function in the NTS. Therefore, we focused on “GABA receptor signaling” in Table 1 and “G protein‐coupled receptor signaling pathway (GO:0007187),” “Plasma membrane region (GO:0098590),” or “Synapse (GO:0045202)” in Table 2, and we chose the GABA_B_ receptor‐mediated signaling pathway and three genes (i.e., *GABBR1*, *ADCY1*, and *CACNB1* genes) arbitrarily.

GABA_B_ receptor is a G protein‐coupled receptor, whereas GABA_A_ receptor is a ligand‐gated ion channel. The binding of GABA or GABA receptor agonist results in the recruitment and activation of Gαi/o proteins. The activated Gαi/o subunits inhibit adenylyl cyclase, resulting in lowered cAMP levels, while Gβγ subunits activate inwardly rectifying potassium channels at postsynaptic sites and inhibit voltage‐dependent calcium channels at presynaptic sites, leading to neuronal inhibition.^28^ In the current study, we observed that the emetic behaviors of the shrew induced the inhibition of the GABA_B_ receptor‐mediated signaling pathway: decreased *GABBR1* gene expression level, increased *ADCY1* gene expression, and decreased *CACNB1* gene expression (Figure 3A). If this observation is true, then its contrapositive is also true. We administered a GABA_B_ agonist to stimulate GABA_B_ receptor‐mediated signaling and observed that no emetic behaviors occurred. The proof by the contrapositive further supported the involvement of the GABA_B_ receptor‐mediated signaling pathway in PONV.

Baclofen, a GABA_B_ receptor agonist, is used for the alleviation of signs and symptoms of spasticity resulting from multiple sclerosis, particularly for the relief of flexor spasms and concomitant pain, clonus, and muscular rigidity.^29^ However, it has not been used for PONV in surgical patients. Previous studies have confirmed that baclofen inhibits emesis. Lei et al. reported that baclofen attenuated the lower esophageal sphincter relaxation in patients with gastroesophageal reflux disease (GERD).^30^ Kawai et al. reported that 40 mg of baclofen oral tablet attenuated esophageal pH decrease in patients with GERD.^31^ Also, Curcic et al. reported that baclofen reduced the frequency of postprandial reflux events in patients with GERD and healthy volunteers.^32^ Kawai et al. reported that baclofen reduces the frequency of vomiting in children with disabilities.^31^ Cohen et al. reported that GABA_B_ receptor agonists improve the symptoms of motion sickness.^33^ One possible explanation for these previous observations is that baclofen stimulated the GABA_B_ receptor‐mediated signaling pathway in the NTS as the vomiting center in the brain and that the efferent outputs from the NTS to induce vomiting were strongly attenuated. Hence, interestingly enough, our findings indicate that baclofen may be useful for prevention and treatment for PONV.

One of the limitations of this study was that we did not measure the nausea sensation of the shrews. Unfortunately, it is difficult to evaluate nausea in animal models owing to our inability to communicate with these animals. However, a recent report suggests that a novel behavior, fruit‐flavored water avoidance, reflects nausea‐associated behaviors of mice.^34^ This methodology may be used in future experiments in shrews.

Another limitation of the current study is that we did not measure protein expression levels in the NTS, such as GABA_B_ receptors or voltage‐dependent calcium channels. However, almost every protein experiment needs a protein‐specific antibody. Although many of these commercially available antibodies show cross‐reactivity among species, including humans, rats, and mice, the reactivity of available antibodies with shrew brain tissues has not been verified. In the current study, we performed some western blotting experiments; however, the primary antibodies did not show reactivity in the shrew brain (data not shown). Differences in the amino acid sequences of proteins among species can result in differences in antibody reactivity, making further molecular experiments difficult.

## 5 CONCLUSIONS

We established the shrew model of PONV. The RNA‐seq quantified the expression level of 52,381 transcripts at the genome‐wide scale. We focused on the relationship between the GABA_B_ receptor‐mediated signaling pathway and PONV. Baclofen, a GABA_B_ receptor agonist, eliminated emetic behaviors in our shrew PONV model. These new findings suggested that baclofen may be a novel therapeutic or prophylactic agent for PONV, particularly for emesis occurring after gynecological surgery.

## CONFLICTS OF INTEREST

The authors declare no competing interests.

## AUTHOR CONTRIBUTIONS

D.K. performed all experiments, analyzed the data, and wrote the draft of the manuscript. S.S. designed the project, conducted the study, interpreted the data, and revised the draft of the manuscript. T.F‐S., K.M., and M.N. helped with the bioinformatic analyses. Y.I‐K. performed the whole‐transcriptome sequencing. Y.I‐K., J.S., and K.K. helped prepare the manuscript and jointly developed the structure and arguments for the article. M.Y. made critical revisions and contributed to the writing of the manuscript. All authors approved the final manuscript.

## Supporting information

Table S1

Video S1

## Data Availability

The FASTQ file data sets of raw sequenced reads in RNA‐seq are available in the DRA (DNA Data Bank of Japan‐Sequence Read Archive) data set repository (accession number: DRA011636, released on May 1, 2021, https://ddbj.nig.ac.jp/DRASearch/).
